# Noise and Robustness in Phyllotaxis

**DOI:** 10.1371/journal.pcbi.1002389

**Published:** 2012-02-16

**Authors:** Vincent Mirabet, Fabrice Besnard, Teva Vernoux, Arezki Boudaoud

**Affiliations:** 1 Laboratoire Joliot-Curie, CNRS, ENS, Université de Lyon, Lyon, France; 2 Laboratoire Reproduction et Développement des Plantes, INRA, CNRS, ENS, Université de Lyon, Lyon, France; University of California, San Diego, United States of America

## Abstract

A striking feature of vascular plants is the regular arrangement of lateral organs on the stem, known as phyllotaxis. The most common phyllotactic patterns can be described using spirals, numbers from the Fibonacci sequence and the golden angle. This rich mathematical structure, along with the experimental reproduction of phyllotactic spirals in physical systems, has led to a view of phyllotaxis focusing on regularity. However all organisms are affected by natural stochastic variability, raising questions about the effect of this variability on phyllotaxis and the achievement of such regular patterns. Here we address these questions theoretically using a dynamical system of interacting sources of inhibitory field. Previous work has shown that phyllotaxis can emerge deterministically from the self-organization of such sources and that inhibition is primarily mediated by the depletion of the plant hormone auxin through polarized transport. We incorporated stochasticity in the model and found three main classes of defects in spiral phyllotaxis – the reversal of the handedness of spirals, the concomitant initiation of organs and the occurrence of distichous angles – and we investigated whether a secondary inhibitory field filters out defects. Our results are consistent with available experimental data and yield a prediction of the main source of stochasticity during organogenesis. Our model can be related to cellular parameters and thus provides a framework for the analysis of phyllotactic mutants at both cellular and tissular levels. We propose that secondary fields associated with organogenesis, such as other biochemical signals or mechanical forces, are important for the robustness of phyllotaxis. More generally, our work sheds light on how a target pattern can be achieved within a noisy background.

## Author Summary

How living organisms affected by natural, stochastic variability achieve regular developmental patterns is a challenging question in biology. A fitting field of investigation is provided by phyllotaxis, the regular arrangements of lateral organs such as leaves or flowers on the stem of vascular plants, as visible on a pinecone or a sunflower head. In spiral phyllotaxis, the most frequent amongst higher plants, these arrangements can be described using spirals, numbers from the Fibonacci sequence and the golden angle, which has led to an ideal, deterministic view of phyllotaxis. Nevertheless, organ initiation can be influenced by cellular and organismal noise. In order to investigate the effect of such noise, and how it might be regulated, we developed a stochastic dynamical model of the inhibitory interactions between organs. Our model predicts stereotypical alterations of phyllotactic patterns, recapitulating disparate observations. Comparing simulations and experiments, we identified the main source of noise affecting phyllotaxis *in planta*. We further propose a generic mechanism of noise regulation through a secondary signal and predict its parameters for an optimal efficiency. More generally, our work suggests that noise can have visible macroscopic effects on developmental phenotypes, and that different layers of control are required to modulate these effects.

## Introduction

**Figure 1:**
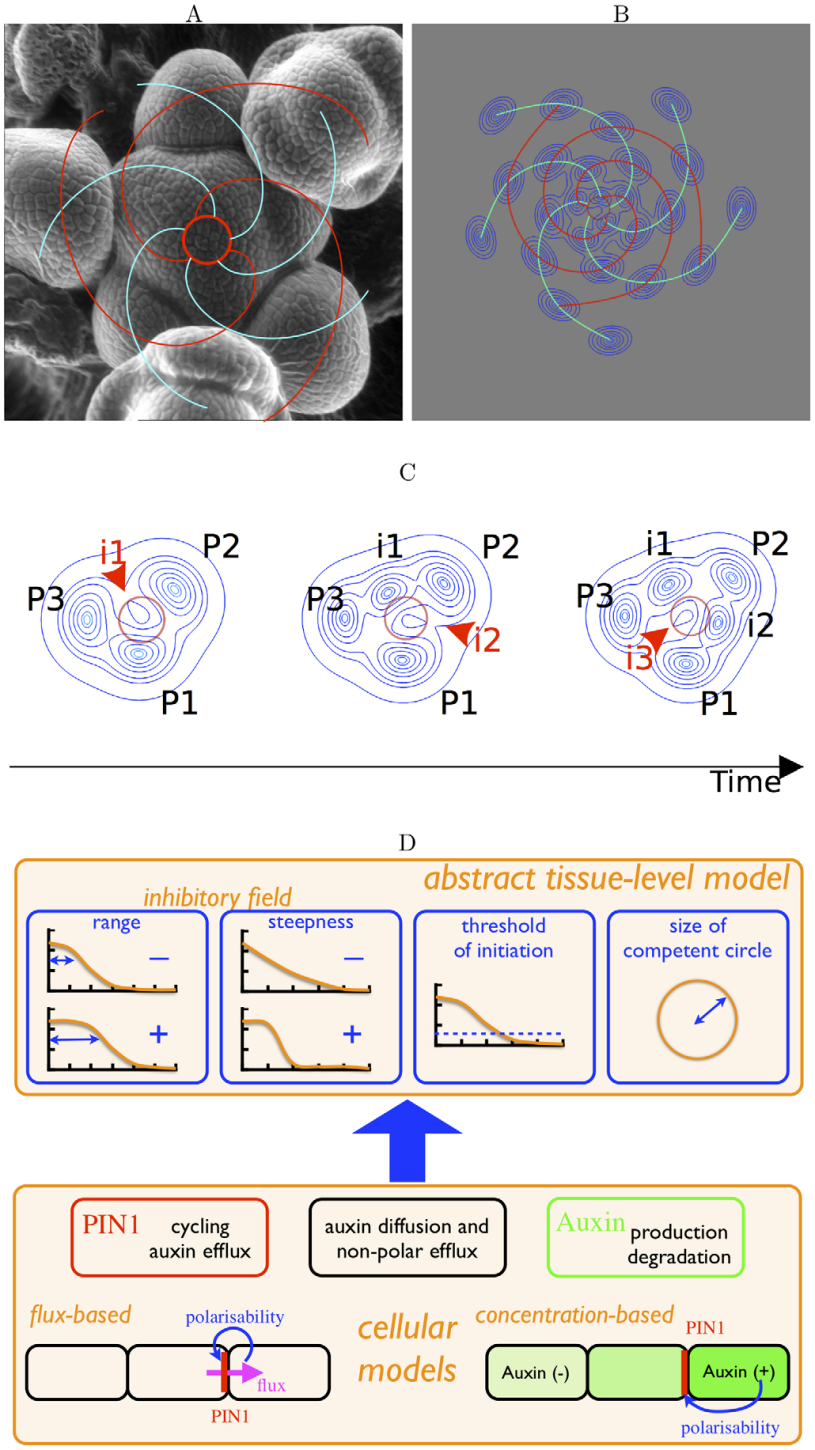
Phyllotaxis, from observations to an abstract model. (A) Scanning electron microscopy picture of the apex of an inflorescence stem of *Arabidopsis thaliana* (ecotype Columbia-0) showing the contact spirals (parastichies); the numbers (3 and 5) of spirals with a given handedness are two consecutive numbers from the Fibonacci sequence. (B) Example of a simulated pattern (using the abstract dynamical model) after 26 primordia have formed (the red circle has the same size as in C). (C) A simulation sequence (also see Video S1). Organ primordia are sources of an inhibitory field (of which the contour lines are shown); primordia are formed on a competent circle (in red) and, because of growth, move away radially; a new primordium (initium) is formed when and where inhibition falls beyond a threshold. (D) A schematic diagram of how the properties of the abstract model emerge from cell-based auxin transport models, see text and supplementary material (in particular Figure S1) for details.

The shoot apex is a major organizer of the aerial architecture of vascular plants. Lateral organs (leaves and flowers) are successively initiated as primordia at the shoot apex yielding a regular arrangement on the stem known as phyllotaxis. Two main categories of phyllotactic patterns are observed: whorled – many primordia emerge simultaneously, and spiraled – a single primordium is initiated at a time. Spiral phyllotaxis features two sets of conspicuous spirals (the parastichies) rotating either clockwise or anti-clockwise, see Figure 1a; the numbers of spirals in each set are often two consecutive numbers of the Fibonacci sequence 1, 1, 2, 3, 5, 8, 13 defined by 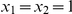, 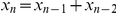; moreover, the angle (viewed from the apex) between two consecutive organs, known as the divergence angle, is often strikingly close to the golden angle, which is about 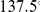. This mathematical beauty has attracted a stream of mathematicians, computer scientists and physicists along with botanists and plant biologists, see for instance [1]–[3] for reviews. A number of models enabled the prediction of spiral phyllotaxis from the interactions between primordia: physical interactions such as optimal packing, e.g. [4] or mechanical forces, e.g. [5], [6], and biochemical interactions such as a reaction-diffusion Turing-like spacing mechanism [7]–[9] or the production of an inhibitor by each primordium preventing initiation in its vicinity [10], [11]. Common to all these studies, phyllotactic spirals are emerge from the self-organization of interacting primordia, as also shown by more abstract dynamical models [12]–[17]. The concept of self-organization was also supported by the observation of phyllotactic-like patterns in physical experiments with ferromagnetic droplets [12], self-assembled solidified microstructures [18], rotating magnets [19], or bubbles floating on a surface [20]. More recently, biological experiments enabled the identification of the primary mechanism of phyllotaxis [21]–[23], i.e. the interaction mechanism behind self-organization. It is now thought that the accumulation of the plant hormone auxin in incipient primordia (initia) through a self-enhancing polar transport creates an auxin depletion playing the role of an inhibitory field, which was further supported by the simulation of cell-based models [24]–[28] in which phyllotaxis emerges from such cell-cell interactions.

Altogether, this body of work is underpinned by an ideal, deterministic view of phyllotaxis, in which perfectly regular patterns can be reproduced by theoretical models. Nevertheless, living organisms are affected by a natural, stochastic variability. Along with a variability among species [1], phyllotaxis proves to be variable at the inter and intra-individual scales [29], [30]. Divergence angles turn out to be widely distributed around the golden angle in *Arabidopsis thaliana* [31]–[34], and almost random in mutants of Arabidopsis [31]–[34] or of rice [29], [35], while short sequences of abnormal divergence angles can occur in sunflower [36] and in Arabidopsis [30]. More generally, a growing attention is given to stochastic variability in organismal development [37], [38]. In plants, stochasticity can be either reflected in development, as in the variability of cell size in the epidermis of sepals [39], or filtered out, as in the robust establishment of the identity of floral organs [40]. This raises the question of how stochasticity impacts on phyllotactic patterns. Auxin cell-based models of phyllotaxis are liable to show a noisy output [24], [26], [27], but their high number of parameters makes them difficult to use for a systematic investigation of the link between variability and its causes [41]. In addition, two classes of models appear in recent literature because the molecular mechanisms controlling the polarization in a given cell of auxin efflux facilitators (PIN-FORMED 1 proteins,abbreviated as PIN1) are largely unknown; these two classes posit polarization according to either the flux of auxin through cell walls (flux-based, [27]) or to the concentration of auxin in neighboring cells (concentration-based, [24], [26], [42]). We therefore chose to use the abstract dynamical system introduced by Douady and Couder in [14], which recapitulates most observed phyllotactic modes, while it enables a comprehensive exploration of the space of parameters. However, in order to make our results relevant to both cellular and tissular levels, we mapped the two cell-based models on this abstract tissue-level model; this mapping can be used to translate cellular parameters into macroscopic phenotypes and, conversely, phyllotactic observations into cellular behaviours. We incorporated stochasticity in this dynamical system and found that stochasticity yields stereotypical alterations of the phyllotactic pattern and that these alterations vary according to the source and intensity of randomness. Finally, inspired by work on noise in the primary patterning of the fruit fly embryo [43]–[46], we investigated whether a secondary inhibitory field could reduce the number of phyllotactic alterations and we predicted the necessary properties of such a field.

## Results

### The inhibitory field model and its correspondence with cellular parameters

We used the dynamical system introduced in [14] which implements the rules stated by Snow and Snow [47]. The main hypotheses are as follows (see Materials and Methods for details). (i) The average stem apex has an axisymmetric shape. (ii) Organ primordia are formed at the periphery of the apex, on a competent circle of radius 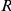, and, because of growth, they move away with a radial velocity 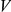, which we assume here to be constant. (iii) Each primordium is a source of inhibition over a region of diameter 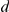 and the steepness 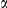 of gradient of inhibition is also a parameter of the model. (iv) A new primordium (initium) is initiated on the competent circle at the location and at the time such that the sum of the inhibitory fields generated by all previous primordia gets below a threshold 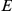. (v) The apex has the shape of a cone and distances are computed on this cone. The angle of the cone was chosen according to the shape of *Arabidopsis thaliana apex*.

A typical simulated spiral sequence is shown in Figure 1c: the inhibition field generated by older primordia in the competent circle decreases as primordia move away, an initium is formed at the place and the time such that inhibition falls below the threshold. This process is repeated leading to a periodic temporal initiation and a spatial establishment of a spiral phyllotactic pattern. Figure 1b illustrates the outcome of this process. A main control parameter of this model is the ratio of the range of inhibition 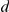 to the radius of the competent circle 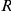 [14], which will be referred to as 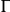. As this ratio is decreased, the phyllotactic mode undergoes a transition from distichous (divergence angles of 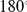) to phyllotactic modes of increasing order [14]: spirals with increasing number of parastichies or whorls with increasing number of simultaneous initiations. Neither the periodicity nor the spatiality of initiation are prescribed; they emerge from the self-organization of the system instead [14].

We next questioned whether this abstract tissue level model could be used to interpret observations at the cellular level. To do so, we re-considered cellular models of auxin polar transport [24], [26], [27]. We sought how the two main classes of cellular models (polarization of auxin efflux based on concentration [24], [26] or based on flux [27]) can be formulated at the tissue level (see Models section and Text S1). Together with previous work [24], [26], [27], [48], our analysis shows that cellular parameters can be mapped on the properties of the abstract model – initiation of primordia close to a circle surrounding the apex, existence of an effective inhibitory interaction between primordia with a well-defined range and steepness, and a threshold for the initiation of a new organ (see Figure 1C and Figure S1). This mapping enables the interpretation of the effect of the abstract model parameters in terms of cellular parameters. Conversely, cell-based scenarii can be translated in parameter sets of the abstract model (Figure S1).

**Figure 2:**
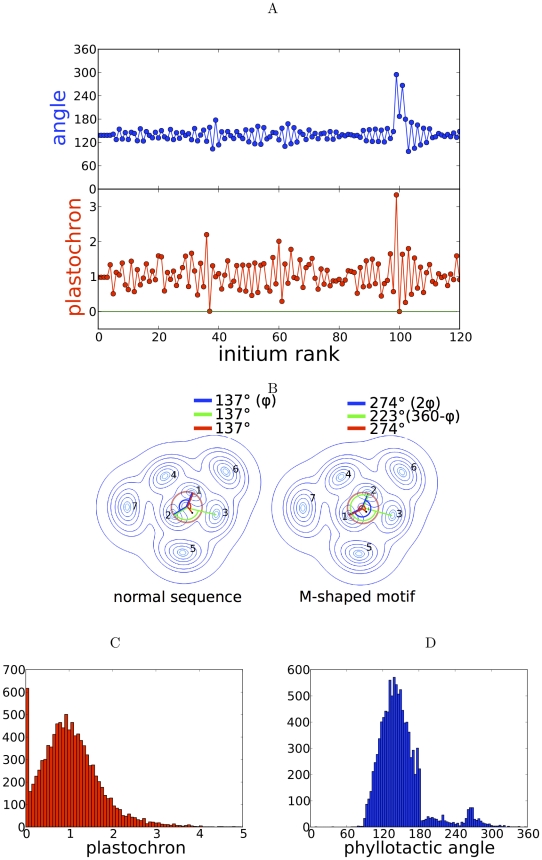
The model with noise on the threshold for organ initiation. (A) A typical sequence of angles and plastochrons (time delay between consecutive initiations); the simulation is started with no noise; M-shaped angle sequences correspond to concomitant initiations (vanishing plastochron). (B) Schematic explaining the origin of M-shaped sequences (same representation as in Figure 1B–E). A vanishing plastochron implies two equivalent initia, which are ranked at random. This either yields a sequence of divergence angles close to the golden angle 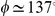 or an M-shaped sequence of the type 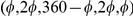. (C) Histograms of plastochrons showing a peak at zero corresponding to concomitant initiations. (D) Histogram of divergence angles (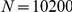) showing a peak close to 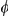, and smaller peaks close to 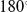 (transient distichous phyllotaxis) and 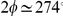 (reflecting M-shaped sequences). Simulation parameters: steepness of inhibition gradient 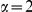, 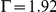, mean threshold for initiation 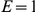, standard deviation of threshold 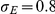.

**Figure 3:**
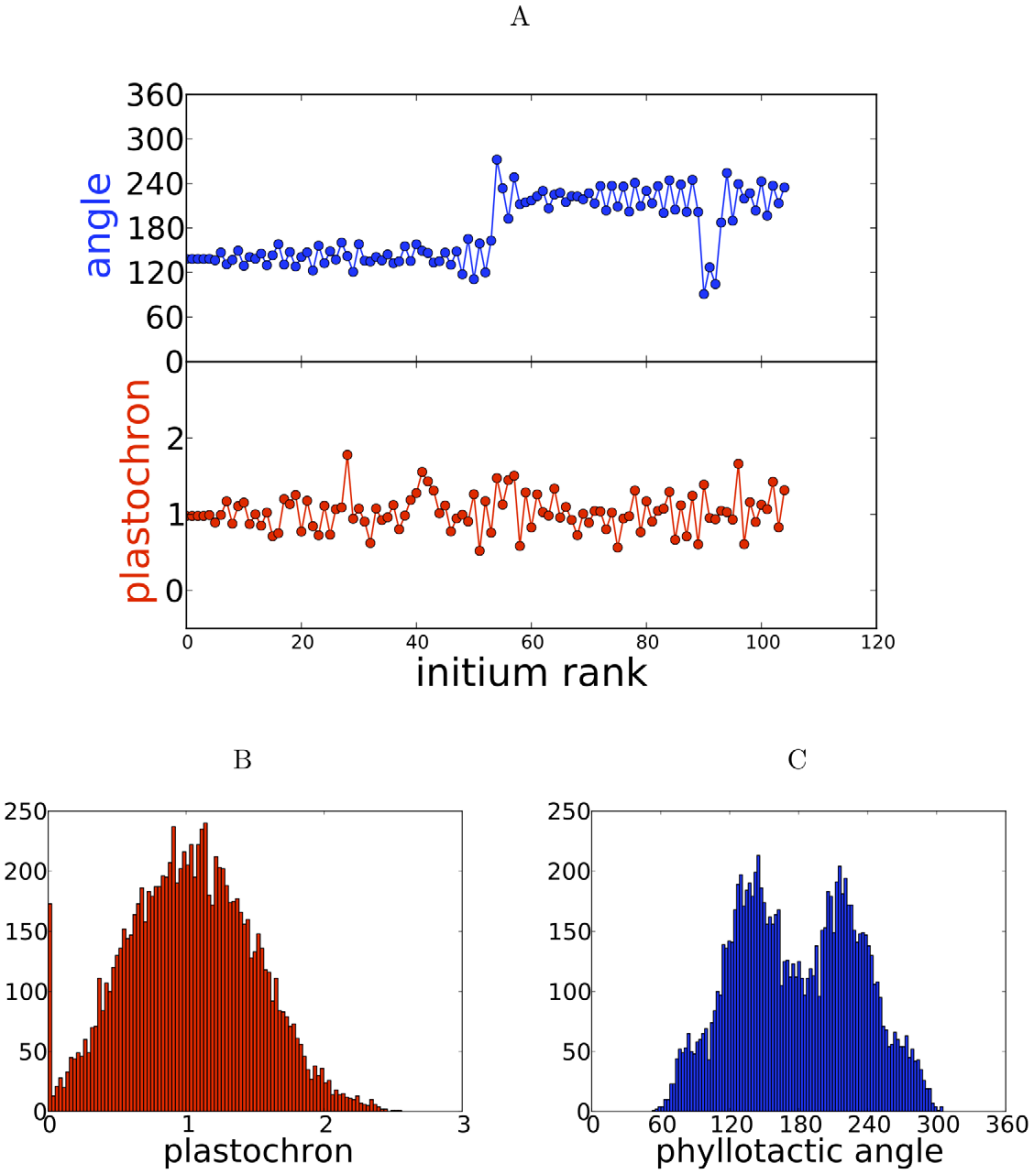
The model with noise on the size of the competent circle. (A) A typical sequence of angles and plastochrons; the simulation is started with no noise; one handedness reversal is clearly visible. (B) Histograms of plastochrons. (C) Histogram of divergence angles (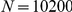) showing peaks corresponding to either handedness: 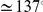 and 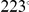. Simulation parameters: steepness of inhibition gradient 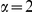, 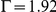, threshold for initiation 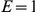, noise strength 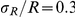.

Accordingly, we subsequently used the abstract dynamical system. In order to investigate the origin of variability of phyllotactic patterns, we incorporated two different sources of noise in this dynamical system (Figures 2 and 3).

### Introducing noise

#### Threshold noise

Organs are initiated when the inhibitory field falls below the threshold 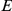. The cellular response to this field can be variable which is equivalent to this threshold being variable. Therefore we first assumed that, before each initiation, the threshold is defined randomly according to a Gaussian distribution. Noise intensity then corresponds to the ratio between the width of the Gaussian and its mean. In order to ensure no confusion between clockwise and anti-clockwise spirals, simulations were started with initial conditions corresponding to the equilibrium of the deterministic model with a left-handed spiral phyllotaxis (parastichies 3–5 as illustrated in Figure 1b). Figure S2 illustrates that constraining the initial conditions does not affect the final results except for ensuring a control of the spiral handedness.

Examples of sequences of angles and plastochrons are shown in Figure 2a. Plastochrons, i.e., the time delay between two initiations events, are subject to fluctuations around a mean corresponding to the value of the deterministic equilibrium of the noise-less model. Plastochron can also vanish, whenever concomitant initiations of two (and more rarely three) primordia occur (see Materials and Methods for a definition of simultaneity). Consequently, the histogram of plastochrons has a maximum at zero delay (Figure 2c).

This raises an issue about organ sorting and more precisely about defining which of the concomitantly initiated organs is the older so as to compute the divergence angle. In view of the equivalence in age between these concomitant organs, we pick their order at random. The concomitant initiation of two organs leads therefore half of the time to characteristic M-shaped patterns, i.e., sequences close to 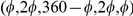, where 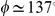 is the golden angle (Figure 2b). Consequently, the histogram of angles has a high peak close to 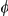 and smaller peaks close to 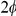 and 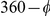 (Figure 2d). It is noteworthy that these M-shaped sequences were observed on sunflower (*Helianthus annuus*) [36] and Arabidopsis (*Arabidopsis thaliana*) [30], reflecting a permutation of the order of organs with respect to a regular sequence.

Consequently, the simple addition of noise in the model can reproduce observed alterations. Another visible alteration is a number of divergence angles close to 180 degrees, corresponding to a transient distichous phyllotaxis. This type of alteration was recently observed in the *plt3 plt5 plt7* multiple mutant of Arabidopsis [32]. In addition to these specific alterations, the sequence of divergence angles shows fluctuations around the golden angle (Figure 2d), as measure in various species [30], [49].

**Figure 4:**
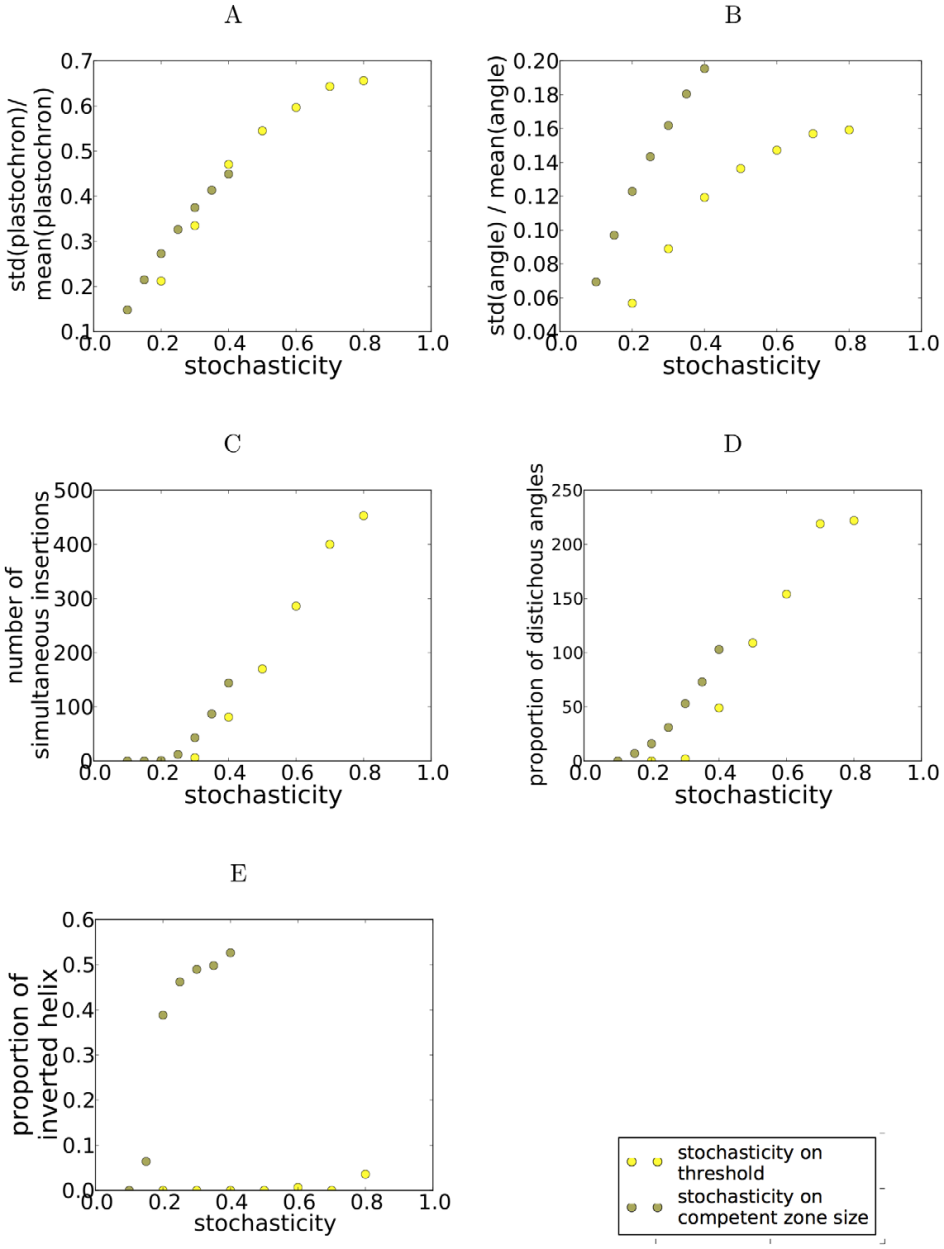
Alterations as a function of noise strength, 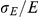 or 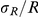 according to the type of noise. (A) Standard deviation of angle normalized by the average angle. (B) Standard deviation of plastochron normalized by average plastochron. (C) The proportion of concomitant initiations. (D) Proportion of distichous angles. (E) Proportion of handedness reversals. Simulation parameters: steepness of inhibition gradient 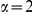, 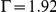, threshold for initiation 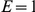.

We then investigated more systematically the space of parameters (also see Figures S3 & S4). The main variations in behavior arise when noise is increased (Figure 4). Low noise only adds a small variability to angles and to plastochrons. Concomitant initiations and transient distichous phyllotaxis appear for a noise strength of about 30%, and increase gradually above. These two alterations are thus the main components of the effect of threshold noise and a change in model parameters only affects their proportion.

#### Size noise

A second possible source of stochasticity lies in the organogenetic activity of the apex, which could be reflected either in the size of the competent circle or in the range of inhibition. As only the ratio, 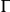, of these two lengths is important, we chose to model a noise in the size of the competent circle: following each initiation, the radius is redefined randomly following a Gaussian distribution. Biologically speaking, this would correspond to a stem cell zone of variable radius, which can be observed in Arabidopsis [50], [51]. Accordingly, noise intensity is quantified as the ratio of the width of this distribution to the radius of the competent circle.

Examples of sequences of divergence angles and plastochrons are shown in Figure 3a. M-shaped angle sequences are again associated with vanishing plastochron, while a few 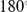 angles also occur indicating a transient distichous mode. In contrast with threshold noise (Figure 2a), an important observation is that the handedness of the spiral is occasionally reversed. This is reflected by a relatively high peak in the histogram of angles around the opposite of the golden angle, i.e., 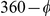. We then investigated systematically the effect of increasing noise intensity on observed patterns (Figure 4). At low intensity, divergence angles and plastochrons have a small variability. At about 16%, handedness reversals and transient distichous patterns appear, and their numbers increase with noise. Concomitant initiations occur above about 25%. We also note that the dependance on noise of the plastochron is similar in the two types of noise; this is not the case for angles, which are more variable with size noise due to the occurrence of handedness reversals. Overall, size noise yields a third type of alterations, handedness reversal; concomitant initiations and distichous angles also occur, but less frequently.

It has been previously argued [14], [36] that M-shaped sequences can arise due to a continuous change in time of 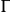, the ratio of inhibition range to competent circle size; this would for instance correspond to the transition from the small vegetative apex to the larger reproductive apex of sunflower. We re-examined these conclusions (Figure S5) and found that fast variations in 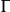 were needed to observe M-shaped patterns or handedness reversals. Therefore, fast fluctuations of 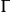 are roughly equivalent to noise on 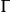.

#### A prediction for the type of noise

As permanent reversals of the handedness of spirals have apparently not been observed [1], [30], we propose that the noise on threshold is more realistic (but see Discussion). In particular threshold noise reproduces the M-shaped sequences of angles that were observed on sunflower [36] and Arabidopsis [30]. In the following, we will assume that noise on threshold is the main source of stochasticity in the system.

### Correcting noise

Available experimental data demonstrate that some mutations make phyllotaxis more irregular than in wild type plants [31]–[35]. This suggests the existence of processes regulating variability in phyllotaxis: either indirectly through e.g. changes in the radius of the competent zone, in the range of inhibition between primordia, modulation of cell activity, or directly by playing on the level of noise. In the latter case, differences between mutant and wild type would be explained within the framework developed above, i.e. a direct regulation of noise intensity. However, previous work in development suggests that it is often more efficient to control variability by adding appropriate feedbacks or additional modules to filter it [38]. In this framework, we sought mechanisms that could regulate variability in phyllotaxis, by adding another layer of control to the model discussed above.

#### Possible scenarios

We categorized the level of controls into three classes. (i) Before initiation. (ii) During initiation. (iii) Post-initiation. In the first class, the outcome would be a reduction in the level of noise introduced in the dynamical system. Auxin signalling could play such a role by filtering fluctuations [52]. In the present framework, this trivially amounts to changing noise strength. In the second and third class, a primordium cannot ‘know by itself’ whether it emerged at the ‘correct’ time and position, because this information is relative to the other primordia. In other words, mechanisms of the last two classes cannot be primordia-autonomous and should also rely on cues coming from older primordia, as the first inhibitory fields does. In a different context, it has been proposed that combinations of morphogens (e.g. diffusible transcription factors) play a role in the regulation of variability during the primary patterning of the fruitfly [43]–[46], but these studies cannot readily be transposed to phyllotaxis. In plants, observations indicate that, in addition to auxin, hormones such as cytokinins [53] or giberellins [54], as well as mechanical forces [55], have an impact on phyllotaxis. It has also been proposed that mechanics cooperates with auxin [42], [56]. Therefore, the existence of other fields playing a role in phyllotaxis is not unrealistic, motivating an investigation of whether a second field can modulate the variability of phyllotaxis.

#### Correction during initiation

So far, auxin is the only hormone that have been shown to be necessary and sufficient for organ formation at the periphery of the shoot apex, see [3]. As auxin is considered as the primary patterning field in phyllotaxis, it seems unlikely that a second field acts at the same level as auxin, i.e. that the field belongs to class (ii). We nevertheless investigated this possibility by defining a second inhibitory field having similar properties to the first, but with different parameters (for details, see Models, and Figures S6, S7). As the initiation of primordia has two inputs, it remains to define how they regulate initiation; two limiting cases can be envisaged: they redundantly repress initiation, or both are needed to synergetically repress initiation. In the redundant case, organs were initiated whenever the sum of the two inhibitions fell below a threshold. This generates many types of equilibria that greatly differ from the Fibonacci spiral (for example divergence angle of 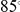 or alternation between values of the divergence angle, Figure S6). We could rationalize this observation as follows: each field favors one phyllotactic mode with a given organ spacing and the two spacings are in general incommensurate as long as the two fields differ in range; the situation is then akin to the impossible matching of two crystals with different spatial periodicities. In the synergetic case, organs were initiated whenever the product of the two inhibitions fell below a threshold. Regular spiral phyllotactic patterns were obtained, however it appeared that the field with the largest range was dominant in determining the equilibrium divergence angle (Figure S7), and the effect of the second field was equivalent to changing permanently the size of the competent circle in the first field. In either case, a second field alters equilibria, and varying its parameters first results in modifying the divergence angle and the plastochron: the resulting sequence lies at a different position in the parameter space. Although we cannot exclude less parsimonious hypotheses, our results suggest that the class of fields acting at the same level (class ii) cannot provide a plausible mechanism to stabilize a regular phyllotactic pattern.

#### Post-initiation noise correction

**Figure 5:**
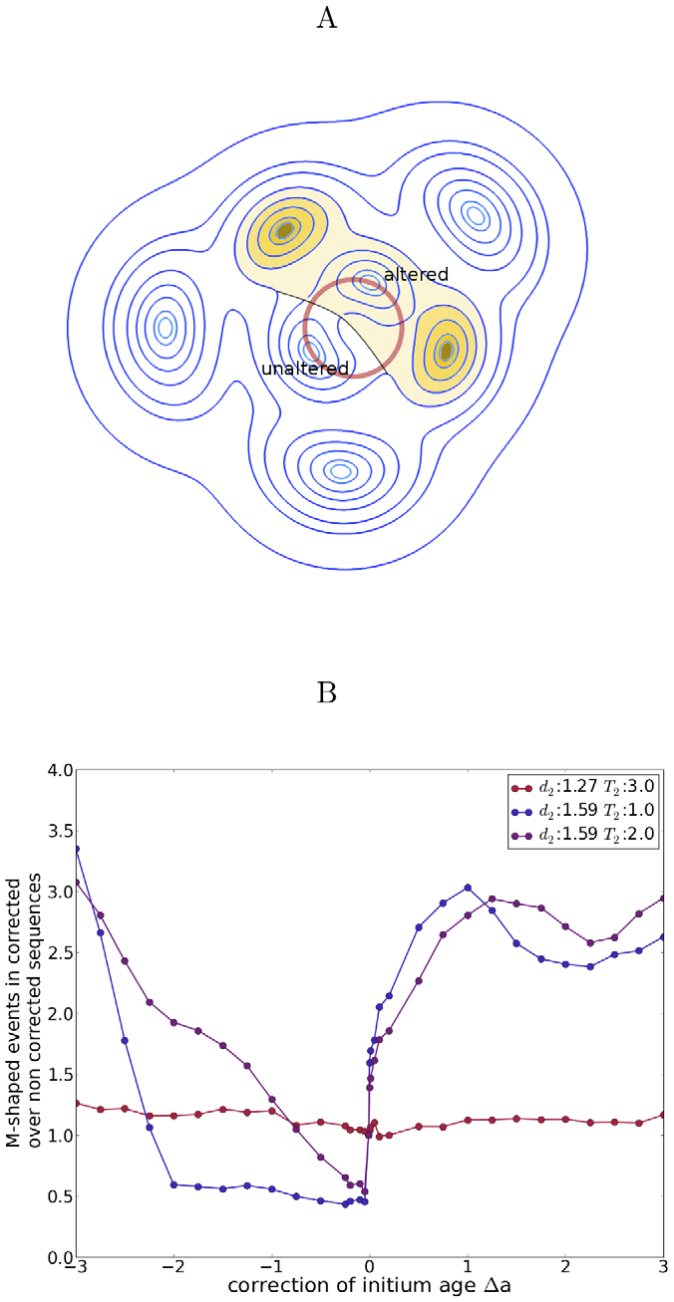
Noise correction by a secondary field. (A) After a time 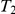 following initiation, each organ generates a secondary field of range 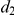 (yellow region for the effect of the two younger primordia). When formed, an initium may be influenced by the secondary field. If so, its age is shifted by an amount 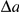. This results in a differential age shift between initia appearing concomitantly. (B) Relative variation in concomitant initiations as a function of the age shift 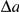 for three points in the parameter space of the secondary field. Note that 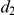 is measured relatively to the range of the primary field (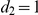 means the same range as the primary field).

We then turned to the third class, where noise correction must act on an intrinsic property of a primordium *after* it has been intiated. In our framework, each primordium is endowed with a spatial position and an age (the time since its initiation). Correcting (by a small amount) the position would only have a small effect on the precision of divergence angles and whence would not remove M-shaped sequences, which were the major alterations in the noise on threshold. Moreover, the M-shaped sequences are due to the incorrect sorting of 2 organs of the same age, so that it is natural to seek whether the age of primordia can be corrected. We focused on a model where the physiological age is changed post-initiation according to the influence of closer primordia. More precisely, we considered a secondary field of range 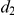, assumed to be steep so that the value of the field is either infinite within its range and 0 beyond (see Materials and methods). Each primordium becomes a source of this secondary field following a time delay 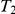 after its initiation. Regarding age correction of initia, we took the simpler form of action: if the secondary field is non zero at the point of initiation, then the physiological age of the initium is incremented with a value 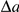, which can be either positive or negative, making this primordium ‘older’ or ‘younger’ respectively (Figure 5a). Indeed it has been shown that the triggering of organogenesis by auxin is modulated by a balance between transcription factors [52], which could make an organ appear earlier or later than when directly prescribed by auxin distribution.

The sequences and histograms of angles were similar to the threshold noise model with no secondary field. However the proportion of alterations differed. We investigated quantitatively the parameter space (see Figure 5b and Figure S8) and found that the secondary field has a significant effect when its range 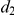 is close to the primary range 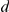 but larger and when the delay 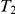 is of the same order of magnitude as the mean time between initia formation (i.e. the average plastochron), more precisely when 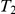 ranges from a fraction of plastochron to two plastochrons. Figure 5b shows the proportion of M-shaped patterns as a function of the increment in age 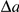. The number of alterations is decreased if the initium is made ‘younger’ when it feels the secondary field (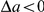), i.e., if its maturation is delayed. For a wide range of values of the shift in age, the number of M-shaped sequences is decreased by a factor of 2. Consequently, a secondary field playing on the differential between organs maturation can significantly improve the regularity of a noisy phyllotactic model.

## Discussion

We investigated the impact of noise on phyllotaxis starting from a deterministic model whereby primordia are sources of an inhibitory field [14], which can be viewed as an abstract representation of the underlying auxin-based dynamics used in more realistic cell-based models (see Text S1 for the mapping). Initia are formed on a competent circle when the inhibitory fields falls below a given threshold and then the primordia move away due to growth. In this model, the temporality and spatiality of organ initiation emerge from the self-organization of the system. This model reproduces most known types of phyllotaxis [14] and we used it in the range of parameters roughly corresponding to the spiral phyllotaxis observed in Arabidopsis, the main parameter being the ratio of the range of inhibition to the radius of competent circle (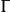). Parameter exploration (Figures S3, S4) only showed differences in the relative intensity of alterations but did not change the overall conclusions.

Most previous theoretical research on phyllotaxis focused on its mathematical regularity. Nevertheless, observations indicate a variability across species and genotypes [1], [29]–[36]. It also appears that studies on cellular models [17], [24], [26], [27] alluded to noise or robustness. Indeed, different sources of stochastic variability can be envisaged, and four of them are discussed hereafter. (i) The discrete nature of the cellular template makes the positioning of an initium according to a given divergence angle only achievable within the precision of a cell radius, as observed in cell-based simulations of auxin transport [24], [26],[27]. The amplitude of the corresponding variability is however generally expected to be small: in the relatively small apex of Arabidopsis cell radius is about 5% of the radius of the competent circle. We did not consider this type of noise because its amplitude (5%) is too small to induce the type of defects presented above. (ii) The level of inhibition can be noisy as recent work suggests that auxin level fluctuates in the shoot apex [52]. (iii) The sensitivity of cells to the signal can be noisy as cellular response can be variable [37], [38]. We integrated these last two sources in a noise on the threshold for initiation. (iv) The activity of the apex can be noisy, which would have an impact on the effective radius of the generating circle [50], [51] and/or the range of inhibition. As only the ratio, 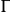, of these two lengths is important, we modeled a noise on the size of the generating circle.

We simulated two sources of noise, on the threshold for initiation and on the size of the generating circle. We found that noise leads to stereotypical alterations, in addition to fluctuations of the divergence angles and plastochrons around their deterministic values: (i) transient distichous pattern with angles of 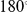; (ii) concomitant initiations corresponding to M-shaped sequences of angles; and (iii) reversal of the handedness of the phyllotactic pattern. These types of alterations correspond to an exploration of phyllotactic modes that are neighbors to the spiral mode: distichous for (i); whorls for (ii); and the spiral with the opposite handedness for (iii). The proportion of the different alterations varies with the source of noise and its strength. M-shaped sequences are visible in sunflower and Arabidopsis [30], [36], angles of 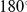 occur in a mutant of Arabidopsis [32], while, to the best of our knowledge, reversals do not happen in nature. A caveat is that long sequences of angles might be required to make sure that a reversal has occurred. A possible explanation for a smaller importance of the noise on size is that the radius of the competent circle (or the range of inhibition) is determined by the behavior of all cells in the apex (respectively the primordium) which leads to some averaging of cellular noise, while the noise on threshold is a local property of the few cells that define an initium. In addition, the number of stem cells might be determined robustly as many levels of regulation are involved [50], [51]. Consequently, a prediction of our work is that the noise on threshold, which corresponds mainly to noise in signaling, is the main source of stochasticity in the Arabidopsis shoot apex.

We then investigated how the noise on threshold might be corrected. A pre-filter simply corresponds to a modulation of the level of noise. Other filters require the propagation of information from older primordia to an initium or to a primordium. Such a transfer of information might be provided by other hormones [53], [54] or by mechanical signals [55]. Therefore we sought whether a second field can reduce alterations: a second field acting on the same level as the first field seems unlikely; a field acting post-initiation could play on the age of primordia. We assumed that each primordium that is old enough is a source of the secondary field and that initia sensing this field have their physiological age shifted. This shift may reflect a slowing down or an acceleration of the initiation of primordia or of the emergence of organs. If the shift is negative (primordia maturation delayed), then the number of M-shaped sequences of angles is significantly reduced. At the cellular level, this shift could be implemented for instance by a delay in the activation of primordia-specific genes or by a decrease in the growth rate of an organ. Our secondary field differs from the one introduced in the dynamical system of [17] to stabilize whorled phyllotaxis, as, there, the two fields have the same spatial dependance. Reaction-diffusion phyllotactic models also used a second field [8] to add memory to the system, which turns out to act as a pre-filter. Our secondary field has more resemblance to proposals made for the early development of the fruitfly embryo [43]–[45] at a smaller scale: the diffusion of the secondary transcription factor Hunchback between nuclei would smooth out the interpretation of the noisy gradient of the primary transcription factor Bicoid. In our case, noise reduction is achieved when an initium is made younger if surrounded by young primordia. Therefore our secondary field implements an averaging of age information between neighboring organs. Our investigation of the space of parameters of our secondary field shows that it is more efficient in noise correction when its range is slightly larger than the range of the primary field and when primordia become sources of the secondary field with a delay ranging from a fraction of plastochron to two plastochrons following their initiation (Figure S8). Indeed mechanical signals, as indirectly reflected by microtubules, seem to become important at about a plastochron following initiation [55]. We predict that other secondary fields should also follow a specific spatial and temporal pattern, in order to be efficient in correction. Although we focused on spiral phyllotaxis, we expect our numerical observations on noise and robustness to also hold for whorled phyllotaxis. It appears that the spatial positioning of organs is rather robust, but that the temporality is more sensitive to noise. Thus secondary fields might be more useful in reducing fluctuations in plastochron.

As we have shown that our model properties can be translated into cellular properties, our results can guide specific cellular simulations that address aspects of stochasticity. Conversely, if the fluctuation of a cellular property can be measured in experiments, it can be translated onto our model using the mapping from cell-based models (Figure S1). Thus our work yields a framework for the analysis of phyllotactic mutants by linking cellular data, the nature of noise, the level of control, and alterations of phyllotaxis. The different layers explored here reflect the complexity of development: inhibitory interactions between primordia emerge from auxin-based cell-cell interactions, phyllotaxis emerges from primordium-primordium inhibitory interactions, a secondary field corrects the phyllotactic pattern by feeding back on cell-behavior. Such a feedback may help achieving a target pattern in a noisy environment and thus provides a general concept in developmental systems biology.

## Models

### Deterministic model

We reimplemented the model of Douady and Couder [14] assuming that (i) the stem has the shape of a cone of angle 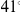 and distances are computed on the cone; (ii) initia are formed on a circle of radius 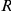 (typical value 2 in arbitrary units) and then move away with a constant radial velocity 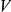 (value 1); (iii) each primordium is a source of inhibitory field that is function of the distance 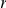 to the primordium,
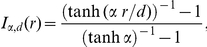


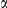
 (typical value 2) measures the steepness of the field and 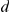 (typical value 3–4) its range, the inhibitory field is the sum of the sources due to all existing primordia; (iv) an initium appears on the competent circle when and where the total inhibition becomes lower than a threshold 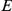 (typical value 1).

The dynamical system was implemented in Python. The time of initiation is found using a standard dichotomic solver. At each step of this process, the minimum of the inhibition on the circle is calculated using the **optimize** library of scipy (http://docs.scipy.org/doc/scipy/reference/optimize.html). The time of initiation is selected whenever this minimum reaches the threshold value. Then an initium is created and the process is repeated. We chose to achieve a precision on time of 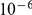 and on space of 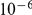 and we checked that these precisions were sufficient to achieve convergence. Simulations were generated on a processor Intel Xeon 2 Ghz.

### Mapping of cellular-based models on the abstract model

We considered the concentration-based model, as formulated by [26], and the flux-based model of [27]. We studied the continuous limit of these cellular models as in [48], [57]; the details are presented in the Text S1.

### Model with noise

We added two sources of noise in the model, on the threshold of initiation and on the radius of the competent circle. In each case, the threshold (resp. the radius) is re-defined, following each initiation, from a random variable of Gaussian distribution of mean 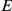 (resp. 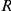) and standard deviation 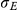 (resp. 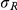).

We investigated the effect of initialization of the simulations by changing the initial values of the divergence angle, turning on noise before or after convergence to a stationary state. We found our results to be unaffected by the type of initialization (Figure S2). Therefore, in order to avoid errors on the measurement of the handedness of the phyllotactic pattern, we started each simulation with initial conditions corresponding to the equilibrium of the deterministic model with a right-handed chirality. Once the simulation had reached a steady state, we turned on noise and started recording the sequences of angles and plastochrons.

When noise was large enough, we frequently observed a vanishing plastochron. Initia were considered as concomitant when the plastochron is smaller than the time-precision of the simulation. In this case, these initia are equivalent and so we defined their order of apparition at random. In order to separate the subsequent M-shaped patterns from other features of variability, we ignored M-shaped patterns when computing the standard deviation of divergence angles, i.e. the standard deviation was computed from the symmetrised distribution of angles with values in (0,180).

### Secondary field

We investigated the effect of a second inhibitory field that is turned on with a delay after initiation and has similar properties to the first inhibitory field.

### Second field of class (ii): During initiation

After a delay 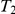 (of the same magnitude as the plastochron) after initiation, each primordium becomes a source of a second inhibition of range 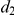 (of the same magnitude as 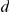), of the same form 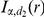 as the first inhibitory field; the second field is the sum of all the contributions of primordia. The new initium will be formed at the point where the interaction between the two fields reaches the threshold 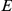. The interactions tested are of redundant type or synergetic type. In the case of redundant inhibition, the total inhibition sensed by a new initium is of the form 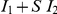 where 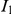 and 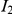 are the two inhibition fields and 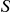 a constant modifying the weight of the second field. In the case of synergetic inhibition, the total inhibition sensed by a new initium is of the form 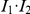 where 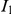 and 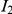 are the two inhibition fields.

### Second field of class (iii): Post-initiation

After a delay 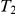 (of the same magnitude as the plastochron) after initiation, each primordium becomes a step-like source of inhibition of range 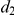 (of the same magnitude as 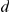), of the form 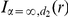 (having the values 0 if 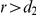 and 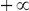 if 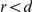); the secondary field is the sum of all the contributions of primordia. Initia are formed according to the primary field, but their age is shifted by a value 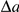 if the secondary field value at the position of inhibition is non zero. Primordia are then ranked according to their corrected age.

## Supporting Information

Figure S1 **Emergence of the abstract model from cell-cell communication.** An arrow, or a bar, indicates that the cellular parameter have a positive, or respectively a negative, effect on the inhibitory field parameter. Auxin parameters concern production, degradation and ‘passive’ diffusion (or non-polar transport), and polar efflux. A different auxin production/degradation serves to define the central zone. Polar efflux parameters concern PIN1 level, PIN1 polarisability (how easily a polar distribution is obtained in response to flux/concentration cues), and efflux efficiency. In the flux-based model, the differentiation of new primordia occurs when an auxin threshold is reached, which directly maps to the threshold of initiation in the abstract model. The parameters of the abstract model are defined in the Main Figure 1C. (A) Concentration-based. (B) Flux-based.
(PDF)

Figure S2
**Effect of noise initialization on convergence of simulations.** (A) Examples of sequences initiated from a distichous state, i.e. a first divergence angle of 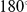, with (blue circles) or with no noise. Each set is composed of a hundred sequences. (B) The distribution of divergence angles according to the type of initialization: a sequence of 100 divergence angles having the value of 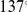, a first divergence angle of 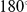 (distichous); noise is turned on either immediately or after 100 plastochrons. This shows that the results are insensitive to initialization. All data are obtained with noise on threshold; simulation parameters: steepness of inhibition gradient 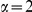, 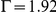, threshold for initiation 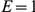, noise strength 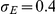.(PDF)

Figure S3
**Role of noise. Exploration of the parameter space of the inhibitory field, as a function of the field steepness** 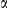 **.** The data plotted are averaged over values of the ratio 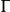 of the inhibition range to the radius of the competent circle in the interval 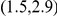. Noise on threshold (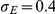) and noise on size (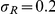). (A) Standard deviation of angle normalized by average angle. (B) Standard deviation of plastochron normalized by average plastochron. (C) Proportion of concomitant initiations. (D) Proportion of distichous angles. (E) Proportion of handedness reversals. (Same definitions as in Main Figure 4.)(PDF)

Figure S4
**Role of noise. Exploration of the parameter space of the inhibitory field, as a function of the value of the ratio** 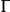 **of the inhibition range to the radius of the competent circle.** The data plotted are averaged over values of the steepness 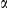 in the interval 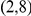. Noise on threshold (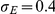) and noise on size (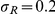), except that 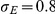 was chosen for D and E in order to reveal alterations. (A) Standard deviation of angle normalized by the average angle. (B) Standard deviation of plastochron normalized by average plastochron. (C) The proportion of concomitant initiations. (D) Proportion of distichous angles. (E) Proportion of handedness reversals. (Same definitions as in Main Figure 4.)(PDF)

Figure S5
**Deterministic model: Role of the decrease in** 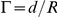 **corresponding to the transition from vegetative to reproductive stage.** A varying 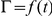 was imposed in 350 time steps for (A,C,E) and 140 time steps for (B,D,F), corresponding to a slow and fast variation respectively. (A–B) Value of 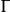 as a function of the initium rank (the number of primordia produced since the beginning of the simulation). (C–D) Divergence angle as function of the initium rank. (E–F) Plastochron as a function of the initum rank. Concomitant initiations mostly occur at small values of 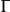 corresponding to high order phyllotaxis with large numbers of parastichies; the fastest decrease of 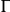 (as in Douady & Couder, 1996) yields more frequent concomitant initiations. A reduction in numerical precision also increases concomitant initiations.(PDF)

Figure S6
**Two fields acting at the same level; redundant interaction.** (A) Phase diagram showing three possible behaviors following the parameters 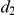 and 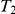, respectively the range of the second field and the delay after which a primordium becomes a source of the second field: I convergence toward the standard equilibrium but with modified 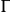, II diverse equilibrium with oscillations, III convergence toward an equilibrium of 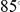. The limits of the phase diagram are only slightly modified by parameter 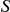. (B,C,D) illustrate sequences of the three categories (B) Category I, 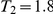 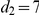 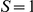 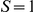 (C) Category II, 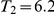, 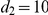, 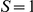 (D) Category III, 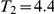, 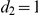, 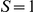
(E) effect of 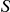 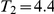, 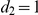, 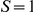
(blue), 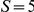
(red). For all panels: steepness of inhibition gradient 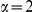, 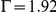, threshold for initiation 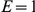, condition for initiation 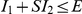. This figure shows that redundant interaction yields unrealistic phyllotactic modes.(PDF)

Figure S7
**Two fields acting at the same level; synergetic interaction.** Divergence angles (in stationary regime) as a function of the range 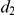 of the second field, the delay 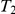 after which a primordium becomes a source of the second field. In this case changing the intensity 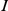 of the second field amounts to changing the initiation threshold 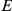 in the simple case with one field. (A,B) Two 3D views. (C) Cuts for 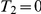 (blue) and 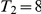 (red); in the latter case, the second field has no effect unless 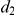 is large enough. Parameters of inhibition (same for the two fields): steepness of inhibition gradient 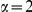, 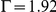, threshold for initiation 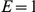, condition for initiation 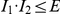. With this regulation, the field with the largest range is dominant; adding a second field acting synergetically with the first one amounts to changing the parameters of the first one (mostly increasing 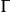).(PDF)

Figure S8
**Exploration of the parameter space of the secondary field.** (A) Number of concomitant initiations as a function of the age shift 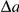 for various parameters. 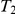 is the time of activation of the secondary field. 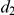 is the size of the secondary field normalized by that of the first. For large 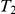, the secondary field has an effect only when its range is large enough. Secondary fields of range smaller than 1 are unefficient. (B) Parameter space illustrating the proportion of new initia overlapped by (i.e. feeling) the second field. Black to yellow color illustrates no overlap to full overlap. The secondary field has an effect only when the overlap is partial (red color): it then generates a differential aging between the two concomitant initia because only one feels the secondary field.(PDF)

Text S1 **Mapping of cellular-based models on the abstract model.**
(PDF)

Video S1 **A temporal sequence of the deterministic model corresponding to** **Figure 1B** **.** Opens in VLC media player.
(AVI)
