# Supplementary figures and images for: Noise and Robustness in Phyllotaxis
Source: PLoS Comput Biol. 2012 Feb 16;8(2):e1002389. doi: 10.1371/journal.pcbi.1002389 (PMC3280957; doi:10.1371/journal.pcbi.1002389)

Figure S1

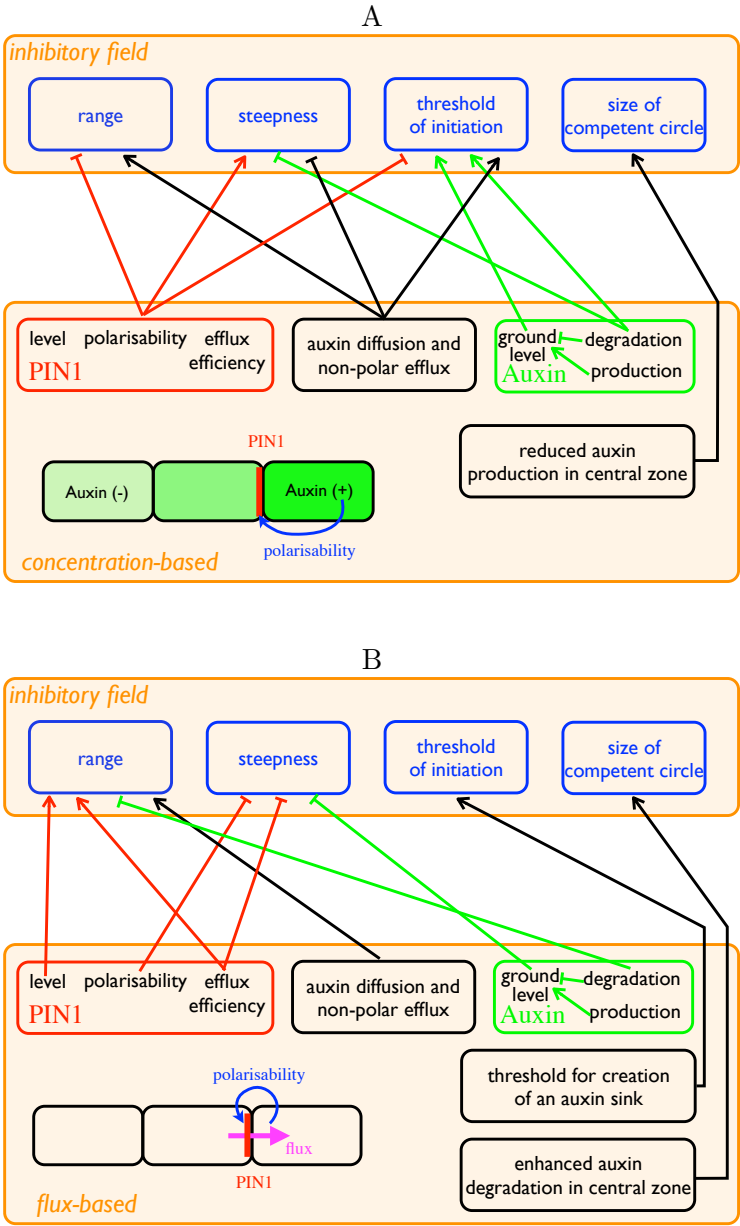

Supplement: Figure S1 — Emergence of the abstract model from cell-cell communication. An arrow, or a bar, indicates that the cellular parameter have a positive, or respectively a negative, effect on the inhibitory field parameter. Auxin parameters concern production, degradation and ‘passive’ diffusion (or non-polar transport), and polar efflux. A different auxin production/degradation serves to define the central zone. Polar efflux parameters concern PIN1 level, PIN1 polarisability (how easily a polar distribution is obtained in response to flux/concentration cues), and efflux efficiency. In the flux-based model, the differentiation of new primordia occurs when an auxin threshold is reached, which directly maps to the threshold of initiation in the abstract model. The parameters of the abstract model are defined in the Main Figure 1C. (A) Concentration-based. (B) Flux-based. (PDF) [file pcbi.1002389.s001.pdf]

Figure S2

A

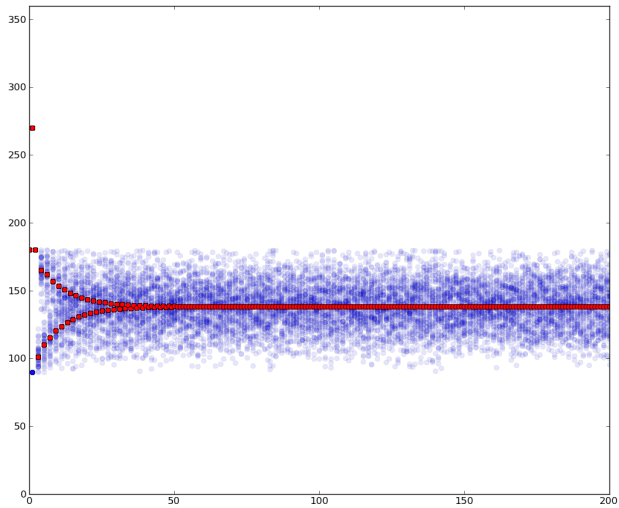

B

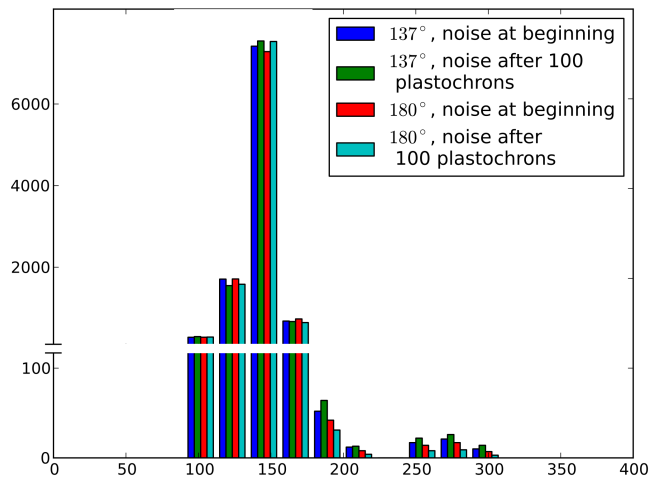

Supplement: Figure S2 — Effect of noise initialization on convergence of simulations. (A) Examples of sequences initiated from a distichous state, i.e. a first divergence angle of , with (blue circles) or with no noise. Each set is composed of a hundred sequences. (B) The distribution of divergence angles according to the type of initialization: a sequence of 100 divergence angles having the value of , a first divergence angle of (distichous); noise is turned on either immediately or after 100 plastochrons. This shows that the results are insensitive to initialization. All data are obtained with noise on threshold; simulation parameters: steepness of inhibition gradient , , threshold for initiation , noise strength . (PDF) [file pcbi.1002389.s002.pdf]

Figure S3

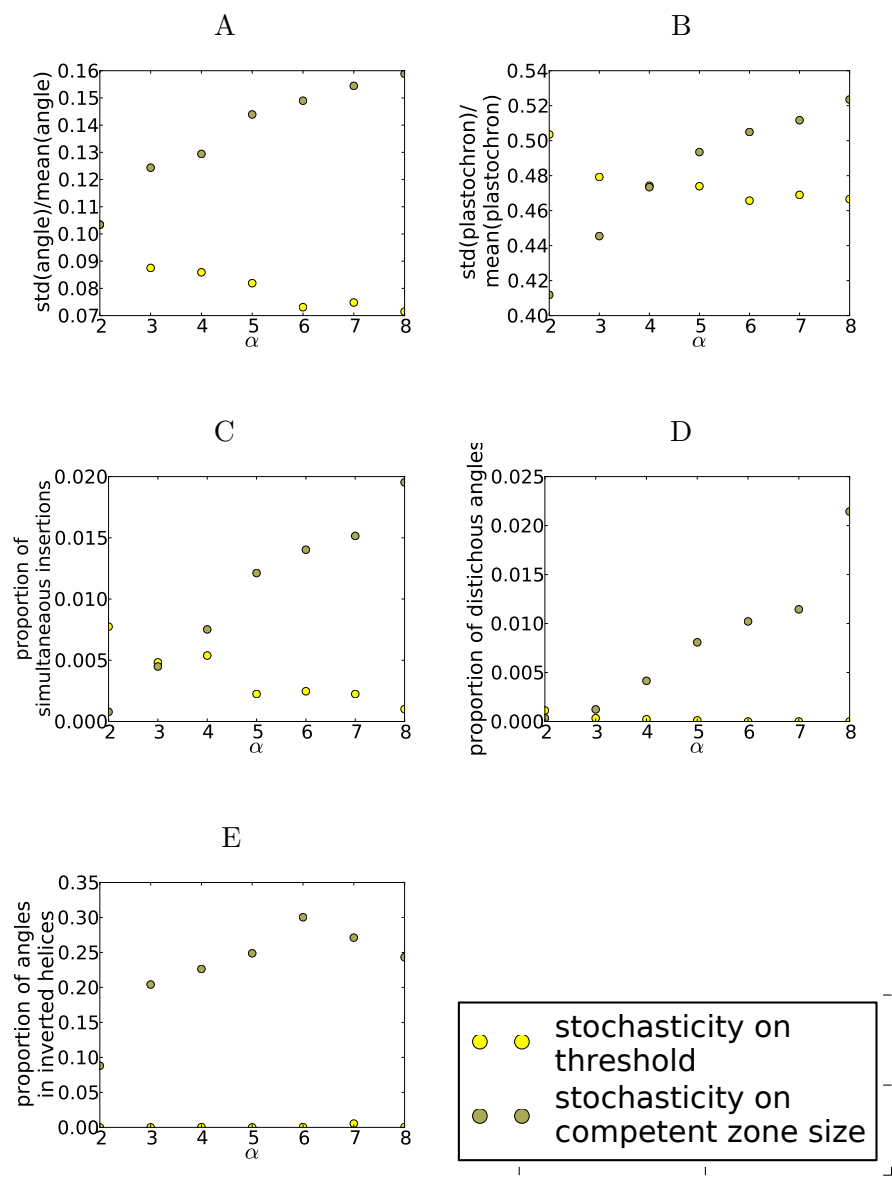

Supplement: Figure S3 — Role of noise. Exploration of the parameter space of the inhibitory field, as a function of the field steepness . The data plotted are averaged over values of the ratio of the inhibition range to the radius of the competent circle in the interval . Noise on threshold () and noise on size (). (A) Standard deviation of angle normalized by average angle. (B) Standard deviation of plastochron normalized by average plastochron. (C) Proportion of concomitant initiations. (D) Proportion of distichous angles. (E) Proportion of handedness reversals. (Same definitions as in Main Figure 4.) (PDF) [file pcbi.1002389.s003.pdf]

Figure S4

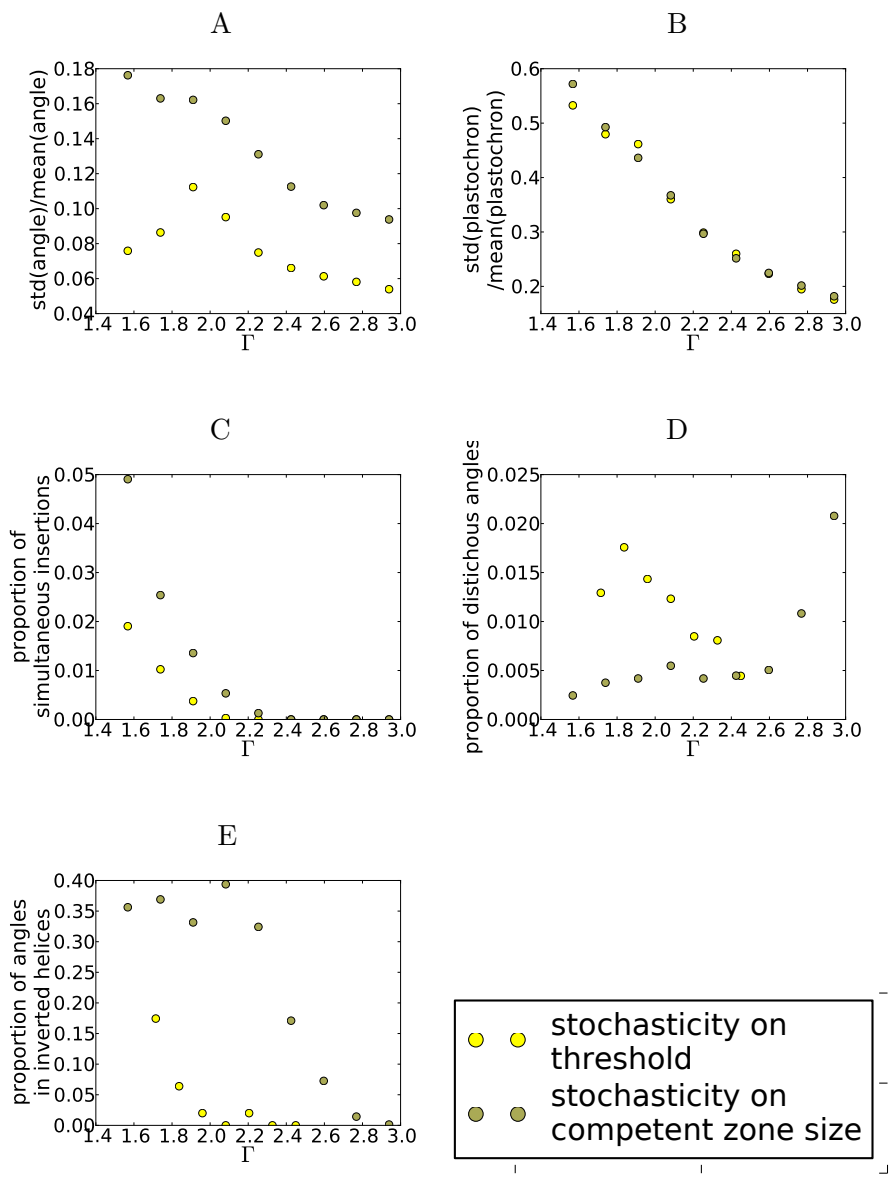

Supplement: Figure S4 — Role of noise. Exploration of the parameter space of the inhibitory field, as a function of the value of the ratio of the inhibition range to the radius of the competent circle. The data plotted are averaged over values of the steepness in the interval . Noise on threshold () and noise on size (), except that was chosen for D and E in order to reveal alterations. (A) Standard deviation of angle normalized by the average angle. (B) Standard deviation of plastochron normalized by average plastochron. (C) The proportion of concomitant initiations. (D) Proportion of distichous angles. (E) Proportion of handedness reversals. (Same definitions as in Main Figure 4.) (PDF) [file pcbi.1002389.s004.pdf]

Figure S5

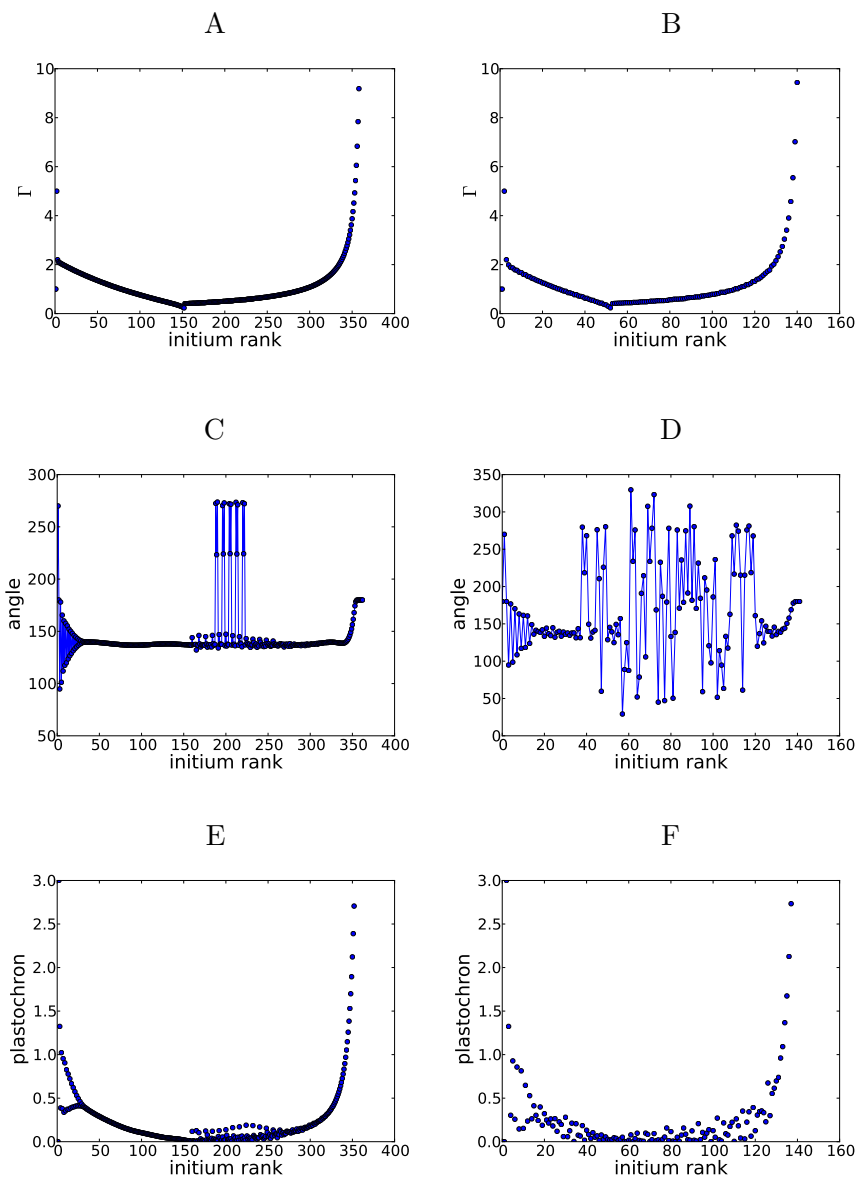

Supplement: Figure S5 — Deterministic model: Role of the decrease in corresponding to the transition from vegetative to reproductive stage. A varying was imposed in 350 time steps for (A,C,E) and 140 time steps for (B,D,F), corresponding to a slow and fast variation respectively. (A–B) Value of as a function of the initium rank (the number of primordia produced since the beginning of the simulation). (C–D) Divergence angle as function of the initium rank. (E–F) Plastochron as a function of the initum rank. Concomitant initiations mostly occur at small values of corresponding to high order phyllotaxis with large numbers of parastichies; the fastest decrease of (as in Douady & Couder, 1996) yields more frequent concomitant initiations. A reduction in numerical precision also increases concomitant initiations. (PDF) [file pcbi.1002389.s005.pdf]

Figure S6

A

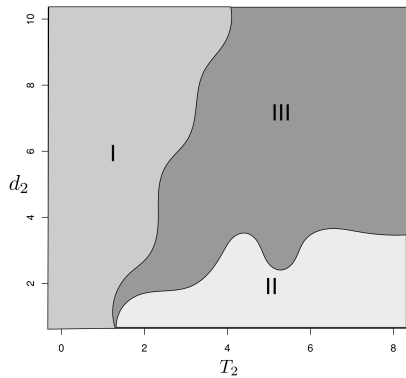

B

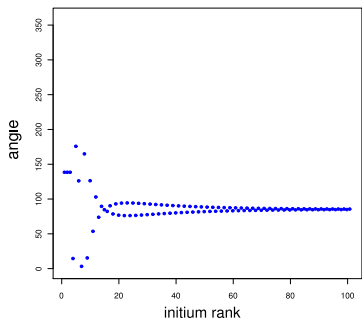

C

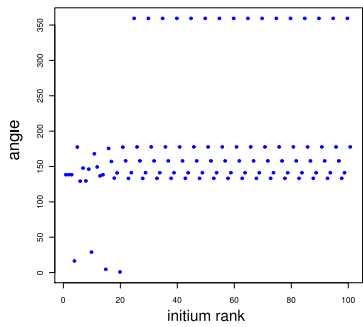

D

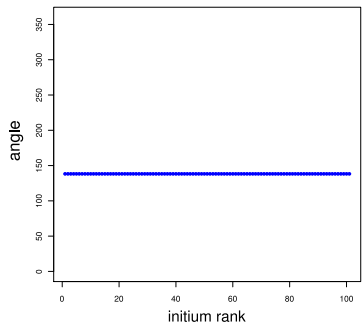

E

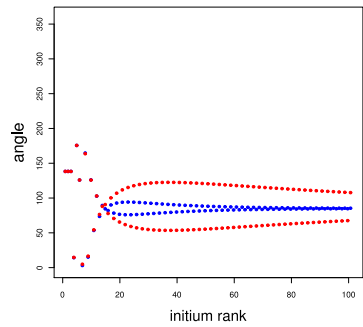

Supplement: Figure S6 — Two fields acting at the same level; redundant interaction. (A) Phase diagram showing three possible behaviors following the parameters and , respectively the range of the second field and the delay after which a primordium becomes a source of the second field: I convergence toward the standard equilibrium but with modified , II diverse equilibrium with oscillations, III convergence toward an equilibrium of . The limits of the phase diagram are only slightly modified by parameter . (B,C,D) illustrate sequences of the three categories (B) Category I, (C) Category II, , , (D) Category III, , , (E) effect of , , (blue), (red). For all panels: steepness of inhibition gradient , , threshold for initiation , condition for initiation . This figure shows that redundant interaction yields unrealistic phyllotactic modes. (PDF) [file pcbi.1002389.s006.pdf]

Figure S7

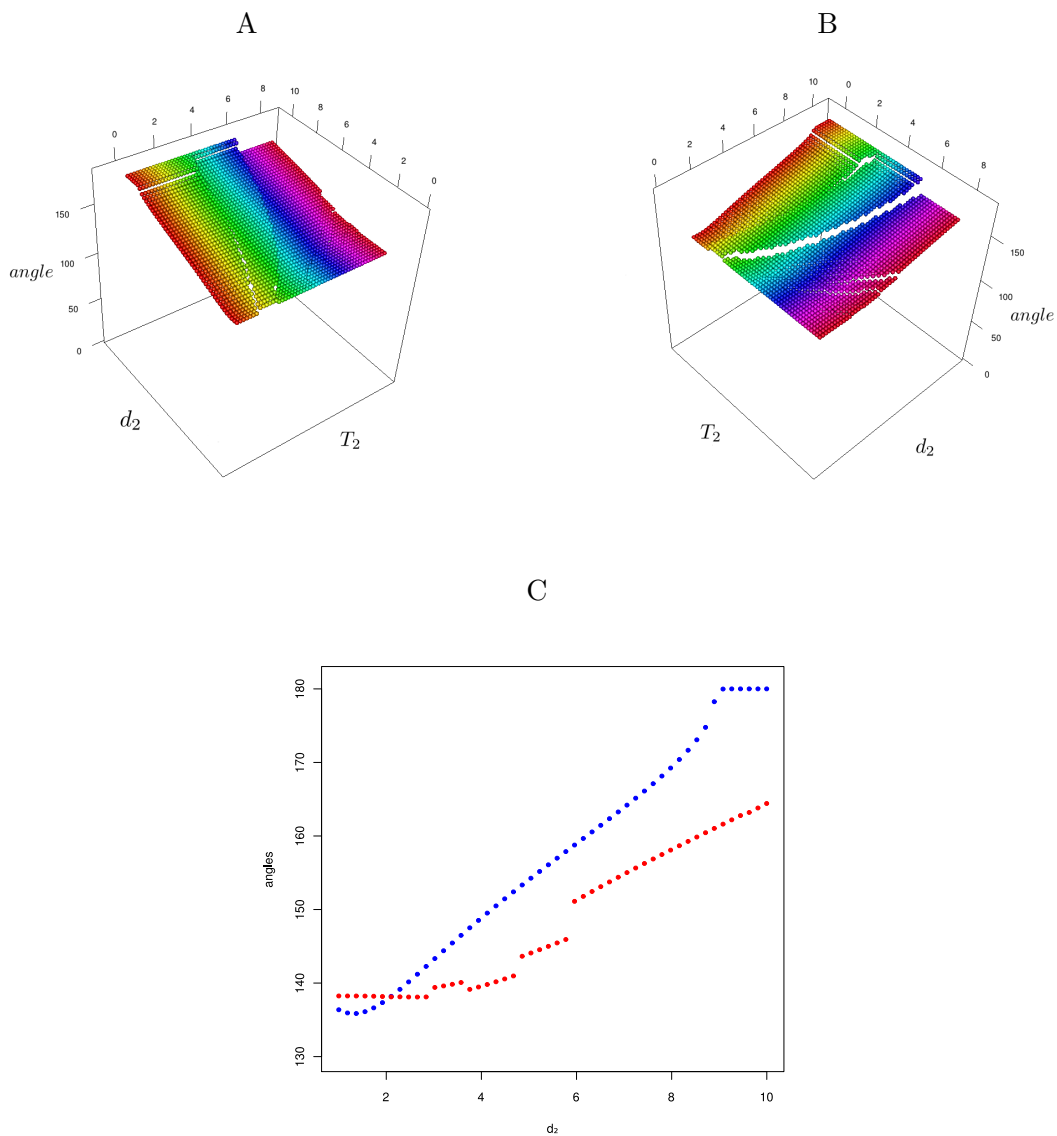

Supplement: Figure S7 — Two fields acting at the same level; synergetic interaction. Divergence angles (in stationary regime) as a function of the range of the second field, the delay after which a primordium becomes a source of the second field. In this case changing the intensity of the second field amounts to changing the initiation threshold in the simple case with one field. (A,B) Two 3D views. (C) Cuts for (blue) and (red); in the latter case, the second field has no effect unless is large enough. Parameters of inhibition (same for the two fields): steepness of inhibition gradient , , threshold for initiation , condition for initiation . With this regulation, the field with the largest range is dominant; adding a second field acting synergetically with the first one amounts to changing the parameters of the first one (mostly increasing ). (PDF) [file pcbi.1002389.s007.pdf]

# Figure S8

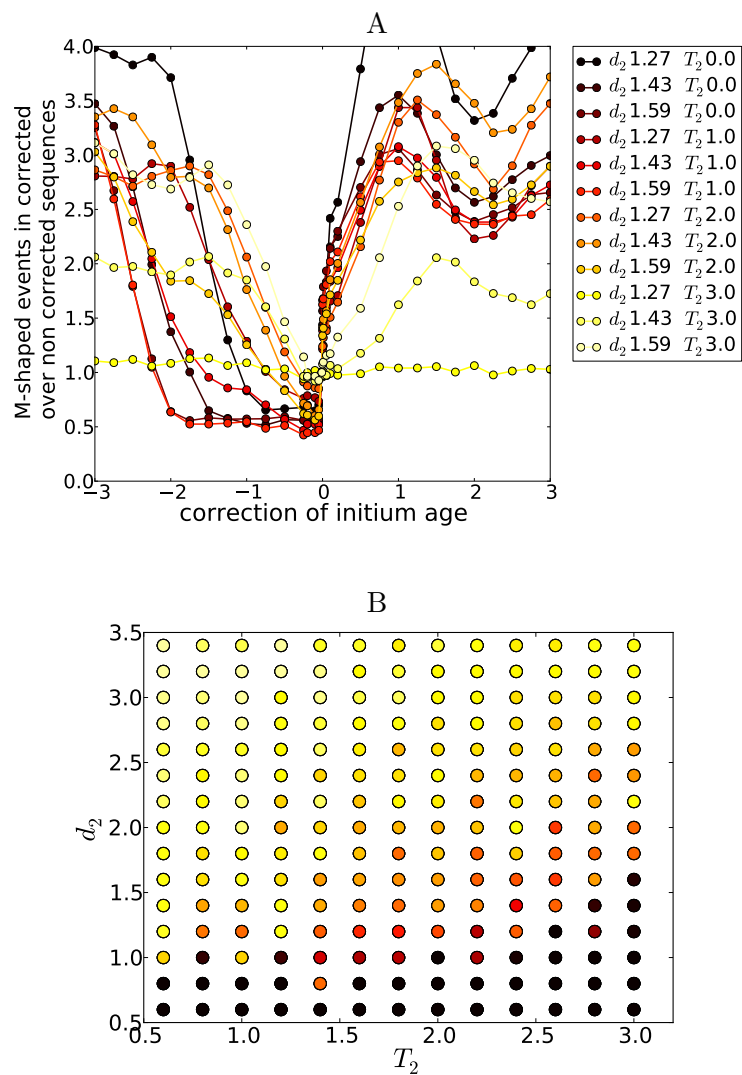

Supplement: Figure S8 — Exploration of the parameter space of the secondary field. (A) Number of concomitant initiations as a function of the age shift for various parameters. is the time of activation of the secondary field. is the size of the secondary field normalized by that of the first. For large , the secondary field has an effect only when its range is large enough. Secondary fields of range smaller than 1 are unefficient. (B) Parameter space illustrating the proportion of new initia overlapped by (i.e. feeling) the second field. Black to yellow color illustrates no overlap to full overlap. The secondary field has an effect only when the overlap is partial (red color): it then generates a differential aging between the two concomitant initia because only one feels the secondary field. (PDF) [file pcbi.1002389.s008.pdf]
