# Supplementary material for: Noise and Robustness in Phyllotaxis
Source: PLoS Comput Biol. 2012 Feb 16;8(2):e1002389. doi: 10.1371/journal.pcbi.1002389 (PMC3280957; doi:10.1371/journal.pcbi.1002389)
Supplement: Text S1 — Mapping of cellular-based models on the abstract model. (PDF) [file pcbi.1002389.s009.pdf]

Noise and robustness in phyllotaxis

Vincent Mirabet[1,2], Fabrice Besnard[2], Teva Vernoux[2,*] Arezki Boudaoud[2,1,*]
1 Laboratoire Joliot-Curie, CNRS, ENS, Université de Lyon, 46 Allée d'Italie, F-69364 Lyon Cedex 07, France
2 Laboratoire Reproduction et Développement des Plantes, INRA, CNRS, ENS, Université de Lyon, 46 Allée d'Italie, F-69364 Lyon Cedex 07, France

Supplementary text

Mapping of cellular models onto the abstract model

Here we analyze how the abstract model of phyllotaxis emerges from the two main classes of cellular models of auxin dynamics. In cellular models, the central zone is defined by an enhanced auxin degradation or a reduced auxin synthesis, while growth results in the movement of cells away from the centre. We start from the cell-level description of auxin dynamics and polarization of the PIN1 auxin efflux facilitator, and obtain a continuous tissue-level description of auxin dynamics, from which we deduce the parameters of the abstract inhibitory field model (Fig. S1).

For simplicity (and with no lack of generality), we consider a one-dimensional line of cells, indexed by an integer i. We note a_i auxin concentration in cell i, p_i^+ (p_i^-, respectively) the PIN1 quantity facilitating efflux from cell i to cell $i+1$ (cell i to cell $i-1$, respectively). Auxin flux from cell i to cell $i+1$ is the sum of a 'diffusive' part (of efficiency γ_{D}) accounting for auxin diffusion between cells or its facilitated influx/efflux by non-polarly distributed proteins, and of an 'active' part (efficiency γ_{A}) accounting for the effux facilitated by the polarly distributed PIN1:

$$J_{i>i+1} = \gamma_{\mathrm{D}}(a_i - a_{i+1}) + \gamma_{\mathrm{A}}(p_i^+ a_i - p_{i+1}^- a_{i+1}). \tag{1}$$

The variation of auxin level in cell i is due to incoming and outcoming fluxes and to auxin production (rate α_a) and degradation (rate β_a):

$$\frac{\mathrm{d}a_i}{\mathrm{d}t} = J_{i-1>i} - J_{i>i+1} + \alpha_a - \beta_a a_i, \tag{2}$$

where $\mathrm{d}/\mathrm{d}t$ is the derivation with respect to time. $a_{\mathrm{eq}} = \alpha_a/\beta_a$ is the equilibrium auxin concentration in the absence of fluxes. Equations (1-2) need to be complemented with equations relating PIN1 concentrations to auxin. Cellular models assume that PIN1 are polarized according to concentrations in neighboring cells [1,2] or to the flux of auxin [3], as will be stated more explicitly below.

Concentration-based

We use the formulation of [2], where auxin in a cell i promotes recruitment of PIN1 in the plasma membranes (of neighbouring cells) that are oriented towards cell i. Assuming PIN1 cycling to be faster than auxin dynamics, this leads to

$$p_i^{\pm} = P\frac{a_{i\pm 1}}{\kappa + (a_{i-1} + a_{i+1})/2}, \tag{3}$$

P being the total PIN1 amount per cell (assumed to be the same for all cells) and κ represents a characteristic difference in auxin concentration for polarization — the ratio of the equilibrium auxin concentration $a_{\rm eq} = \alpha_a/\beta_a$ to κ measures cell polarizability.

The continuous limit of this model was obtained in [4], assuming that auxin gradients occur over many cells and that auxin fluctuations are not too large. The principle is to introduce a continuous auxin field $a(x,t)$ such that $a_i(t) = a(i\ell, t)$, x being the spatial coordinate and ℓ cell length, and to expand equations (1-3) in powers of ℓ. This leads to a partial differential equation for $g(x,t) = a(x,t) - a_{\rm eq}$ [4],

$$\frac{\partial g}{\partial t} = -H\frac{\partial^2 g}{\partial x^2} - M\frac{\partial^4 g}{\partial x^4} - \beta_a g - K_1\frac{\partial}{\partial x}\left(g\frac{\partial g}{\partial x}\right) - K_2\frac{\partial}{\partial x}\left(\frac{\partial g}{\partial x}\frac{\partial^2 g}{\partial x^2}\right), \tag{4}$$

where all additional parameters are related to the cell-level parameters:

$$\begin{aligned} H &= \ell^2(\gamma_{\rm A} P a_{\rm eq}^2/(\kappa + a_{\rm eq})^2 - \gamma_{\rm D}), \\ M &= \ell^4 \gamma_{\rm A} P a_{\rm eq}^2/(\kappa + a_{\rm eq})^2/4, \\ K_1 &= 2\ell^2 \gamma_{\rm A} P \kappa a_{\rm eq}/(\kappa + a_{\rm eq})^3, \\ \text{and } K_2 &= \ell^4 \gamma_{\rm A} P a_{\rm eq}^2/(\kappa + a_{\rm eq})^3. \end{aligned}$$

When polar transport is sufficiently more efficient than diffusion, more precisely when $H^2 > 4M\beta_a$, then the uniform solution $g(x,t) = 0$ (or $a(x,t) = a_{\rm eq}$) is unstable with respect to small perturbations. This is the regime leading to phyllotactic positioning of auxin maxima [1, 2, 4]. The analysis of Equation (4) was performed in depth in [4]; we recall and derive all results needed for the mapping to the abstract model. The spacing of auxin maxima corresponds to the range of inhibition in the abstract model, and is given by $d = 2\pi\sqrt{2M/H}$, which extends over a few cells, and decreases with the efficiency of polar transport. When auxin variations are small, an approximate solution of Eq. (4) takes the form

$$\begin{aligned} g(x,t) &= A\cos(2\pi x/d) + B\cos(4\pi x/d), \text{ with} \\ A &= \frac{2M\sqrt{(H^2 - 4\beta M)\,(2H^2 + \beta M)}}{H\sqrt{(HK_2 - 2K_1 M)\,(HK_2 + K_1 M)}}, \quad B = -\frac{M\left(H^2 - 4\beta M\right)}{H\left(HK_2 + K_1 M\right)}. \end{aligned}$$

This form enables finding the other parameters of the abstract model. The threshold of inhibition roughly corresponds to the ratio of the smallest auxin level $(a_{\rm eq} - A - B)$ to the largest level $(a_{\rm eq} + A - B)$. Finally, the shallowness of the inhibitory field is directly given by the ratio B/A; the larger this ratio, the steeper the inhibitory field.

We used the correspondences established above to draw in Fig. S1A the links between the cellular, concentration-based model, and the abstract inhibitory field model.

Flux-based

We use the formulation of [3], where outcoming flux through a plasma membrane promotes recruitment of PIN1 to this membrane. Assuming PIN1 cycling to be faster than auxin dynamics, this leads to

$$p_i^+ = \left(\kappa\ \phi(J_{i>i+1}) + \alpha_p\right)/\beta_p, \tag{5a}$$

$$p_i^- = \left(\kappa\ \phi(-J_{i-1>i}) + \alpha_p\right)/\beta_p, \tag{5b}$$

where $\phi(x) = x$ if $x > 0$ and else $\phi(x) = 0$; κ measures the strength of the feedback of flux on PIN1, and the ratio κ/β_p measures the polarizability of PIN1 distribution; α_p is the rate of incorporation of PIN1 to the membrane, while β_p is the rate of endocytosis of PIN1, so that $P = \alpha_p/\beta_p$ is the ground level of PIN1 in a membrane.

Following the same methodology performed in [4] for the concentration-based model, we obtained the continuous limit of this model, assuming that auxin gradients occur over many cells. The principle is to introduce a continuous auxin field $a(x,t)$ such that $a_i(t) = a(i\ell, t)$, x being the spatial coordinate and ℓ cell length, and to expand equations (1,5) in powers of ℓ. This leads to a partial differential equation for $a(x,t)$,

$$\frac{\partial a}{\partial t} = \frac{\partial}{\partial x}\left(\frac{D}{1 - a/a_c}\frac{\partial a}{\partial x}\right) + \alpha_a - \beta_a a. \tag{6}$$

This equation appears as a nonlinear diffusion equation (note that $a < a_c$) with parameters

$$D = (\gamma_{\mathrm{D}} + \gamma_{\mathrm{A}} P)\,\ell^2, \quad a_c = \frac{\beta_p}{\gamma_{\mathrm{A}}\kappa},$$

and an equilibrium auxin concentration $a_{\mathrm{eq}} = \alpha_a/\beta_a$.

The parameters of the abstract inhibitory field have direct correspondences with the equations above. The range of inhibition corresponds to the characteristic length of auxin variations $d = \sqrt{D/\beta_a}$ when auxin level is small (close to an auxin sink). In the model of [3], an initium is created when auxin reaches a level higher than a threshold ω; this threshold of activation is exactly the mirror image of the threshold of inhibition in the abstract model. Finally, the steepness of auxin gradient is measured by the ratio a_c/a_{eq}; the larger this ratio, the steeper the gradient.

We used these correspondences to draw in Fig. S1B the links between the cellular, flux-based model, and the abstract inhibitory field model.

References

1. Smith R, Guyomarc'h S, Mandel T, Reinhardt D, Kuhlemeier C, et al. (2006) A plausible model of phyllotaxis. P Natl Acad Sci Usa 103: 1301–1306.

2. Jonsson H, Heisler M, Shapiro B, Meyerowitz E, Mjolsness E (2006) An auxin-driven polarized transport model for phyllotaxis. P Natl Acad Sci Usa 103: 1633–1638.

3. Stoma S, Lucas M, Chopard J, Schaedel M, Traas J, et al. (2008) Flux-based transport enhancement asa plausible unifying mechanism for auxin transport in meristem development. Plos Comput Biol 4: e1000207.

4. Newell AC, Shipman PD, Sun Z (2008) Phyllotaxis: cooperation and competition between mechanical and biochemical processes. J Theor Biol 251: 421–39.
